# Productivity loss among people with early multiple sclerosis: A Canadian study

**DOI:** 10.1177/13524585211069070

**Published:** 2022-02-09

**Authors:** Elisabet Rodriguez Llorian, Wei Zhang, Amir Khakban, Scott Patten, Anthony Traboulsee, Jiwon Oh, Shannon Kolind, Alexandre Prat, Roger Tam, Larry D Lynd

**Affiliations:** Collaboration for Outcomes Research and Evaluation (CORE), Faculty of Pharmaceutical Sciences, The University of British Columbia, Vancouver, BC, Canada; School of Population and Public Health, The University of British Columbia, Vancouver, BC, Canada/Centre for Health Evaluation and Outcome Sciences (CHÉOS), St. Paul’s Hospital, Vancouver, BC, Canada; Collaboration for Outcomes Research and Evaluation (CORE), Faculty of Pharmaceutical Sciences, The University of British Columbia, Vancouver, BC, Canada; Department of Psychiatry, University of Calgary, Calgary, AB, Canada; Division of Neurology, Department of Medicine, The University of British Columbia, Vancouver, BC, Canada; Division of Neurology, St. Michael’s Hospital, University of Toronto, Toronto, ON, Canada; Division of Neurology, Department of Medicine, The University of British Columbia, Vancouver, BC, Canada; Department of Neurology, Faculty of Medicine, Université de Montreal, Montreal, QC, Canada; Department of Radiology and School of Biomedical Engineering, The University of British Columbia, Vancouver, BC, Canada; Collaboration for Outcomes Research and Evaluation (CORE), Faculty of Pharmaceutical Sciences, The University of British Columbia, Vancouver, BC, Canada/Centre for Health Evaluation and Outcome Sciences (CHÉOS), St. Paul’s Hospital, Vancouver, BC, Canada

**Keywords:** Multiple sclerosis, work productivity loss, unpaid productivity loss, fatigue

## Abstract

**Objectives:**

To analyze work productivity loss and costs, including absenteeism (time missed from work), presenteeism (reduced productivity while working), and unpaid work loss, among a sample of employed people with multiple sclerosis (pwMS) in Canada, as well as its association with clinical, sociodemographic, and work-related factors.

**Methods:**

We used cross-sectional data collected as part of the Canadian Prospective Cohort Study to Understand Progression in MS (CanProCo) and information from the Valuation of Lost Productivity questionnaire.

**Results:**

Among 512 pwMS who were employed, 97% showed no or mild disability and 55% experienced productivity loss due to MS in the prior 3 months. Total productivity time loss over a 3-month period averaged 60 hours (SD = 107; 23 from presenteeism, 19 from absenteeism, and 18 from unpaid work), leading to a mean cost of lost productivity of CAD$2480 (SD = 4282) per patient, with an hourly paid productivity loss greater than the wage loss. Fatigue retained significant associations with all productivity loss outcomes.

**Conclusion:**

Unpaid work loss and productivity losses exceeding those of the employee alone (due to teamwork and associated factors) are key additional contributors of the high economic burden of MS. Workplace accommodations and treatments targeted at fatigue could lessen the economic impact of MS.

## Introduction

Multiple sclerosis (MS), a chronic disease of the central nervous system with variable severity and disability duration,^1^ not only impacts health and well-being but also represents a major economic burden.^2,3^ Since MS affects people in their most productive years of life (typically, diagnosis occurs between 20 and 40 years of age),^4^ productivity loss has been found to be the main cost driver for most severe cases of the disease.^2,5^

Typically, productivity loss due to illness comprises absenteeism (time missed from work) and presenteeism (reduced productivity while working) for people who are employed, as well as unpaid work productivity loss (from activities such as housework, shopping, or childcare) for all people.^6^ However, previous studies have applied a wide variety of definitions and instruments.^7^ Notably, common practice is to use respondents’ income to quantify costs of lost time attributable to presenteeism and absenteeism,^8–12^ and unpaid work losses have been ignored from existing MS productivity loss monetary valuations. While the use of income fails to account for additional costs resulting from team productivity loss and other job and workplace features,^13^ failing to account for unpaid work loss can further underestimate the burden of MS.

In Canada, even though indirect costs have been identified as a major component of MS costs,^14–16^ last available estimates are based on data that are almost a decade old^16^ and only considered productivity loss associated with absenteeism by accounting for sick leave and retirement due to MS. The objective of this study was to characterize work productivity loss and costs in a sample of employed Canadians with MS, as well as its association with a set of clinical, sociodemographic, and work-related factors.

## Methods

### Data and design

We used baseline, cross-sectional data collected between January 2019 and April 2021 as part of the Canadian Prospective Cohort Study to Understand Progression in MS (CanProCo). CanProCo is a 5-year prospective cohort study conducted in five sites across four Canadian provinces (Alberta, British Columbia, Quebec, and Ontario) with the primary aim to better understand MS disease progression. CanProCo obtained local research ethics board approval before study initiation, and all participants provided written informed consent. Details on CanProCo inclusion criteria, ethics, and informed consent are provided as supporting information (S1).

### Productivity loss

Productivity loss components were measured using the Valuation of Lost Productivity questionnaire (VOLP), previously validated and applied in other diseases.^13,17^ The key outcomes of interest for this study, all measured for the last 3 months, were (1) paid work productivity loss (hours) due to absenteeism; (2) paid work productivity loss (hours) due to presenteeism; (3) unpaid work productivity loss (hours); and (4) total cost of lost productivity (the sum of the cost of paid and unpaid work productivity losses).

To calculate the total cost of lost productivity (i.e. attaching a monetary value to time loss), different aspects of each individual’s work environment including team size, contribution to team productivity, and availability of perfect substitutes were used to obtain wage multipliers. Costs of paid work lost productivity were calculated as “time lost × hourly wage × multiplier.” As for costs of unpaid work loss, we used hourly earnings of CAD$15.60 reported by Statistics Canada for home childcare and home support workers.^18^ Additional details on measuring productivity loss and costs are provided in S1.

### Variables associated with productivity loss

We evaluated the association between productivity loss and costs with sociodemographic, clinical, quality of life, and work-related characteristics based on previous research.^19–21^ Sociodemographic variables included sex and age. In terms of clinical predictors, the severity of disease was measured using the Expanded Disability Status Scale (EDSS) which ranges from 0 to 10 in 0.5 increments, which indicate a higher level of disability. The Modified Fatigue Impact Scale (MFIS)^22^ that contains physical, cognitive, and psychosocial items was used to measure fatigue; the Patient Health Questionnaire (PHQ)-9^23,24^ was used for depression and the seven-item Generalized Anxiety Disorder (GAD-7) questionnaire^25^ for anxiety, with higher scores signaling greater levels of distress. Other clinical variables included in the analysis were time since diagnosis in years; whether the patient was using a disease-modifying therapy (DMT); number of comorbidities; whether the patient had a relapse in the past 3 months; and MS phenotype. We also included health-related quality of life utility using health states from the EQ-5D-5L instrument^26^ and associated value set for Canada,^27^ as well as work habits (usually sitting, standing, or walking during the day; lifting either light or heavy loads) and type of employment (full-time, part-time, and self-employed).

### Statistical analysis

The analysis centered on those participants who were employed at the time information was collected. Given the zero-inflated and skewed nature of the data, we evaluated the association of all productivity loss outcomes with the selected group of variables using two-part models. The model was first composed of a logistic regression for the probability of observing a positive-versus-zero productivity loss outcome, followed by a generalized linear model (GLM) with log link and gamma distribution, fitted for those participants showing non-zero (i.e. some) productivity loss. To improve the interpretation of the coefficients from the two-part models, we generated a marginal (or incremental) effect of each factor on productivity loss.^28^ To determine which factors to include in the multivariate analysis, univariate two-step models were first created. Only those variables with a *p* value ⩽0.1 in the resulting univariate analysis joint test of significance^28^ were included in the final multivariate two-part model. Furthermore, given the high statistical correlation (see S2) and conceptual overlap between considered distress variables (fatigue, depression, and anxiety),^29^ the multivariate model only included the MFIS indicator of physical, cognitive, and psychosocial fatigue.

## Results

### Study cohort and patient characteristics

**Figure 1.**
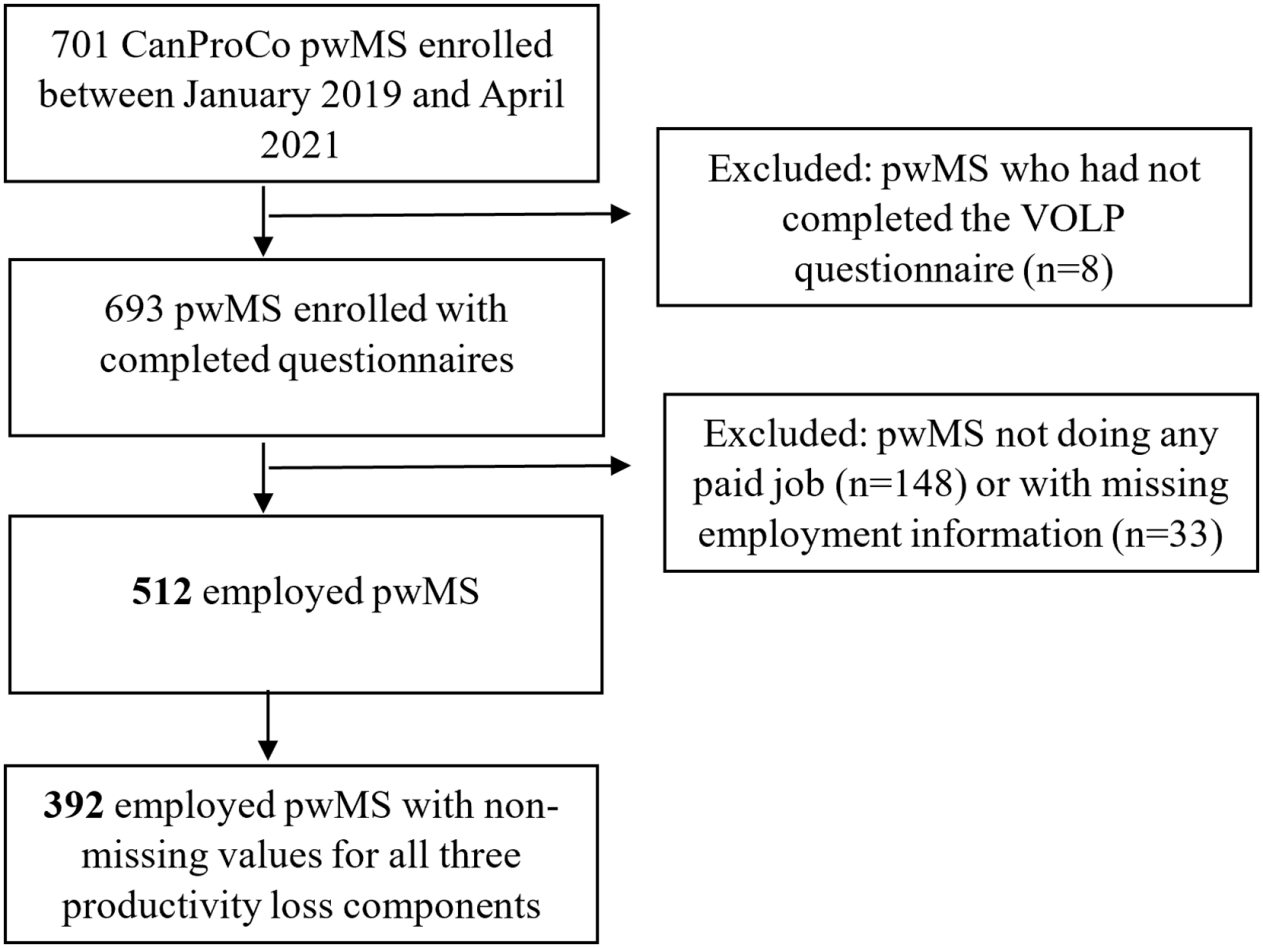
Study cohort.

Figure 1 presents the study sample selection process. From a total of 693 pwMS enrolled in the CanProCo study who had completed the required questionnaires by April 2021, 512 (74%) were working for pay, 148 (21%) were not doing any paid work, and 33 (5%) did not specify their employment status. Of those employed, 72% were working full-time, 16% part-time, and 12% were self-employed.

As shown in Table 1, the sample of 512 employed pwMS was mostly female (71%) with RRMS (83%) and mild disability (EDSS 1–3.5: 72%). The mean age of participants was 39 (SD = 9.5) years, and the mean duration of MS was 3.4 (SD = 2.7) years. In addition, 56% of participants were receiving a DMT, 7% had a recent relapse, and 37% declared having no comorbidities, while 20% had more than three comorbidities. Most jobs required participants to be mostly sitting (53%), while 31% had jobs that required them to stand and/or walk, and 16% had occupations that required some type of lifting. Among the 512 eligible employed pwMS, 392 had no missing information for all three productivity loss components. A comparison between employed pwMS depending on whether they had at least one missing productivity loss component shows no substantial differences (see S3).

**Table 1.** Characteristics of employed pwMS.

	Variable	N^a^	Statistic
Sociodemographic	Sex, % female	512	364 (71%)
	Age (years), Mean (SD)	512	38.74 (9.51)
Clinical	Severity		
	No disability EDSS 0	510	128 (25%)
	Mild disability EDSS 1–3.5	510	367 (72%)
	Moderate disability EDSS 4–6	510	15 (3%)
	Time since diagnosis (years), mean (SD)	511	3.35 (2.72)
	MS type, % by phenotype		
	RRMS	512	426 (83%)
	PPMS	512	27 (5%)
	RIS	512	29 (6%)
	CIS	512	30 (6%)
	Current DMT users, %	512	284 (56%)
	Relapsed in the past 3 months, %	475	35 (7%)
	Comorbidities, %		
	0	512	191 (37%)
	1	512	139 (27%)
	2	512	84 (16%)
	+3	512	98 (20%)
	Fatigue, median (max–min)^b^	492	24 (0–81)
	Depression, median (max–min)^c^	503	5 (0–26)
	Anxiety, median (max–min)^d^	500	4 (0–21)
Quality of life	EQ-5D utility score, mean (SD)	508	0.86 (0.10)
Work-related characteristics	Work habits, %		
	Usually sits	497	262 (53%)
	Stand/walk	497	152 (31%)
	Light/heavy lifting	497	83 (16%)
	Employment status, %		
	Full-time	512	366 (72%)
	Part-time	512	84 (16%)
	Self-employed	512	62 (12%)

CIS: clinically isolated syndrome; DMT: disease-modifying therapy; EDSS: Expanded Disability Status Scale; EQ-5D: EuroQol-5D; MS: multiple sclerosis; RRMS: relapsing-remitting MS; PPMS: primary-progressive MS; RIS: radiologically isolated syndrome; SD: standard deviation.

a Respondents with non-missing information included in the analysis of each variable (out of a total of 512 employed pwMS).

b Measured using Modified Fatigue Impact Scale, score ranging from 0 to 84.

c Measured using Patient Health Questionnaire-9, index ranging from 0 to 27.

d Measured using seven-item Generalized Anxiety Disorder questionnaire with a possible maximum score of 21 points, cut points of 5, 10, and 15 might be interpreted as representing mild, moderate, and severe levels of anxiety, respectively.

### Productivity loss

Table 2 shows a characterization of productivity loss and work-related variables. The average working time among participants was 5 days, 37 hours/week. Wage multipliers for absenteeism and presenteeism were 1.43 and 1.38, respectively, indicating an hourly work productivity loss greater than the wage loss.

**Table 2.** Work and productivity-related characteristics.

Variable	*N* (%)	Mean (SD)
Work hours per week	432	36.73 (10.69)
Work days per week	508	4.73 (1.03)
Average annual income	474	62,310.13 (27,644.31)
Multiplier for absenteeism	399	1.43 (0.92)
Multiplier for presenteeism	434	1.38 (0.79)
Total work productivity loss, hours (past 3 months)^a^	392	59.65 (106.52)
Paid loss—absenteeism	392	18.96 (52.37)
Paid loss—presenteeism	392	22.72 (51.91)
Unpaid loss	392	17.97 (61.68)
Non-zero total work productivity loss, hours^b^	214 (55)	109.27 (124.02)
Paid work productivity loss due to absenteeism, hours	461	25.97 (72.31)
Non-zero absenteeism^b^	202 (44)	59.28 (99.90)
Proportion of time loss^c^	406	0.07 (0.17)
Paid work productivity loss due to presenteeism, hours	408	21.83 (51.07)
Non-zero presenteeism^b^	96 (24)	92.76 (67.27)
Proportion of time loss^c^	363	0.05 (0.12)
Unpaid work productivity loss, hours	512	20.54 (75.64)
Non-zero unpaid work loss^b^	94 (18)	111.88 (145.28)
Total costs of lost productivity with multiplier, CAD (past 3 months)	392	2479.75 (4282.43)
Total costs without multiplier, CAD (past 3 months)	392	1848.29 (3171.79)
Non-zero costs of lost productivity^b^	214 (55)	4542.35 (4924.62)

The difference in the number of respondents included in the analysis of each variable (*N*) was due to missing responses for some variables.

CAD: Canadian dollar; SD: standard deviations.

a Statistics presented under this heading are calculated among pwMS with non-missing values for all three productivity loss components.

b Statistics correspond to those pwMS showing a non-zero productivity loss.

c Calculated as the proportion of time loss from regular work time.

Fifty-five percent of participants experienced at least some productivity loss in the past 3 months. In addition, 44% of participants missed work for health reasons (absenteeism) and 24% reported being able to complete the same work in less time had they not had any health problems (presenteeism). Overall, absenteeism and presenteeism accounted for 7% and 5% of participants’ regular work time, respectively. Average total productivity lost over a 3 month period was 60 hours (SD = 107; 23 from presenteeism, 19 from absenteeism, and 18 from unpaid work) among the 392 pwMS with non-missing values for all three productivity loss components, leading to a mean value of lost productivity of CAD$2480 (SD = 4282) per patient. By only using wages, the mean monetary cost was lower by CAD$632.

Differences in productivity time lost across key variables (namely, disease type, severity, and sex) are shown in S4. There are sharp differences between severity levels; pwMS with an EDSS > 0 showed higher productivity loss for all components, on average. Interestingly, while those with no disability (EDSS = 0) showed higher hours lost attributable to absenteeism than to presenteeism, the opposite happened for those with some level of disability. Among all MS phenotypes, PPMS showed the highest total productivity loss. As for sex, females showed higher losses across all three categories.

### Factors associated with productivity loss

Table 3 shows which variables were found to be associated with each productivity loss outcome and thus were incorporated into the multivariate two-part model (Table 4). Neither sex nor work characteristics were found to be associated with any productivity loss outcome in univariate analysis.

**Table 3.** Factors associated with productivity loss—unadjusted association (marginal effect).

Variable	Absenteeism	Presenteeism	Unpaid work productivity loss	Total costs of lost productivity
**Sociodemographic**				
Female	−4.37 (−19.76, 11.03)	3.71 (−7.14, 14.57)	7.40 (−4.87, 19.66)	557.59 (−313.13, 1428.30)
Age	−**0.16 (**−**0.85, 0.54)**	0.54 (−0.02, 1.11)	**0.43 (**−**0.22, 1.08)**	**18.88 (**−**27.93, 65.68)**
**Clinical**				
Severity	4.09 (−2.21, 10.38)	**9.79 (5.16, 14.43)**	**11.51 (5.31, 17.70)**	**721.88 (337.69, 1106.06)**
Time since diagnosis	−**5.37 (**−**8.00**, −**2.75)**	0.42 (−1.39, 2.23)	**2.44 (**−**0.13, 5.01)**	−102.79 (−253.53, 47.94)
MS phenotype				
RRMS	11.35 (−5.33, 28.02)	−10.21 (−29.89, 9.47)	1.01 (−21.50, 23.51)	159.57 (−1259.71, 1578.85)
PPMS	5.85 (−37.51, 49.21)	14.11 (−25.55, 53.77)	18.91 (−29.34, 67.15)	1518.73 (−1556.24, 4593.70)
RIS	−**17.14 (**−**28.82**, −**5.46)**	−14.52 (−29.14, 0.10)	−16.36 (−27.56, –5.15)	−1412.44 (−2984.14, 159.27)
CIS	Ref.	Ref.	Ref.	Ref.
Current DMT use	4.04 (−8.80, 16.89)	6.99 (−2.81, 16.80)	**12.36 (0.07, 24.66)**	580.03 (−251.35, 1411.40)
Relapse	**49.64 (**−**1.38, 100.66)**	−**18.18 (**−**25.73**, −**10.62)**	7.82 (−13.98, 29.62)	**2467.81 (**−**1190.31, 6125.93)**
Number of comorbidities				
0	Ref.	Ref.	Ref.	Ref.
1	−5.99 (−21.90, 9.92)	−0.02 (−13.21, 13.16)	0.81 (−21.15, 22.77)	−204.10 (−1386.95, 978.75)
2	**−11.36 (−23.78, 1.05)**	5.04 (−10.95, 21.03)	**5.94 (−14.30, 26.18)**	−26.24 (−1150.82, 1098.34)
3+	**12.65 (−7.98, 33.29)**	**15.24 (−1.52, 32.01)**	**20.77 (−0.66, 42.19)**	**1645.69 (334.20, 2957.18)**
Fatigue index MFIS	**0.44 (0.08, 0.80)**	**1.01 (0.72, 1.29)**	**0.89 (0.43, 1.36)**	**93.31 (67.98, 118.64)**
Depression index PHQ-9	**1.42 (0.22, 2.62)**	**3.10 (2.02, 4.18)**	**2.89 (1.34, 4.44)**	**306.83 (217.62, 396.04)**
Anxiety index GAD-7	**1.31 (0.16, 2.46)**	**2.68 (1.68, 3.68)**	**2.33 (0.81, 3.85)**	**232.93 (146.34, 319.52)**
**Quality of life**				
EQ-5D utility score	**−3.20 (−8.49, 2.09)**	**−11.64 (−16.99, −6.29)**	**−11.52 (−18.56, −4.48)**	**−1040.78 (−1457.77, −623.79)**
**Work characteristics**				
Work habits				
Usually sits	6.18 (−10.60, 22.97)	2.07 (−10.57, 14.70)	15.73 (−4.90, 36.36)	311.06 (−803.88, 1425.99)
Stand/walk	5.66 (−14.33, 25.64)	−3.75 (−17.75, 10.24)	10.50 (−15.67, 36.66)	−417.54 (−1555.99, 720.91)
Light/heavy loads	Ref.	Ref.	Ref.	Ref.
Employment status				
Full-time	**35.70 (20.76, 50.64)**	**21.63 (9.83, 33.43)**	5.62 (−13.02, 24.26)	**2301.83 (1454.32, 3149.34)**
Part-time	**59.45 (0.91, 117.99)**	13.03 (−26.59, 52.66)	18.96 (−14.30, 52.22)	**1888.27 (−622.48, 4399.01)**
Self-employed	Ref.	Ref.	Ref.	Ref.

Bold values indicate a joint *p* value ⩽0.1.

CIS: clinically isolated syndrome; DMT: disease-modifying therapy; EDSS: Expanded Disability Status Scale; EQ-5D: EuroQol-5D; GAD-7: seven-item Generalized Anxiety Disorder; MFIS: Modified Fatigue Impact Scale; MS: multiple sclerosis; PHQ: Patient Health Questionnaire; PPMS: primary-progressive MS; RIS: radiologically isolated syndrome; RRMS: relapsing-remitting MS.

**Table 4.** Factors associated with productivity loss—adjusted association (marginal effect).

Variable	Absenteeism	Presenteeism	Unpaid work productivity loss	Total costs of lost productivity
Age	**0.21 (−0.53, 0.94)**	—	0.20 (−0.32, 0.72)	**1.08 (−46.43, 48.59)**
Severity	—	**4.72 (0.21, 9.23)**	**5.90 (0.88, 10.93)**	185.12 (−201.54, 571.78)
Time since diagnosis	**−5.32 (−7.93, −2.72)**	—	1.55 (−0.80, 3.90)	—
MS phenotype				
RRMS	18.00 (2.02, 33.97)	—	—	—
PPMS	2.68 (−38.87, 44.23)	—	—	—
RIS	−14.75 (−28.09, −1.42)	—	—	—
CIS	Ref.			
Current DMT use	—	—	6.37 (−4.56, 17.31)	—
Relapse	**39.33 (−0.07, 78.74)**	**−16.87 (−24.47, −9.26)**	—	**2850.56 (−701.05, 6402.18)**
Number of comorbidities				
0	Ref.	Ref.	Ref.	Ref.
1	−12.70 (−25.31, –0.09)	−5.14 (−15.43, 5.14)	−3.69 (−16.94, 9.56)	−670.18 (−1616.95, 276.59)
2	**−12.45 (−25.23, 0.33)**	3.94 (−9.59, 17.47)	3.78 (−14.11, 21.68)	−285.43 (−1330.38, 759.53)
3+	2.42 (−17.56, 22.41)	0.29 (−12.21, 12.79)	7.07 (−10.18, 24.32)	**175.65 (−849.40, 1200.71)**
Fatigue index MFIS	**0.62 (0.18, 1.05)**	**0.96 (0.64, 1.29)**	**0.64 (0.27, 1.01)**	**94.59 (61.32, 127.87)**
EQ-5D utility score	1.86 (−4.67, 8.38)	3.39 (−3.33, 10.12)	−2.11 (−8.17, 3.95)	285.52 (−297.00, 868.03)
Employment status				
Full-time	**35.80 (18.82, 52.79)**	**18.13 (8.37, 27.89)**	—	**2190.24 (1332.93, 3047.54)**
Part-time	**60.94 (−14.60, 136.49)**	1.95 (−23.75, 27.64)	—	1895.71 (−1485.38, 5276.80)
Self–employed	Ref.	Ref.	—	Ref.

Bold values indicate a joint *p* value ⩽0.1.

CIS: clinically isolated syndrome; DMT: disease-modifying therapy; EDSS: Expanded Disability Status Scale; EQ-5D: EuroQol-5D; MFIS: Modified Impact Scale; MS: multiple sclerosis; PPMS: primary-progressive MS; RIS: radiologically isolated syndrome; RRMS: relapsing-remitting MS.

After multivariate adjustment, each additional point in the EDSS scale (signaling higher severity) averaged an additional 5 hours (95% confidence interval (CI): 0.21, 9.23) of presenteeism and 6 hours (95% CI: 0.88, 10.93) of unpaid work. Absenteeism, on the other hand, was found not to be associated with severity. Notably, fatigue was consistently significantly associated with all productivity loss outcomes. Specifically, each one unit increase in the MFIS index (i.e. increasing fatigue) resulted in an average increase in lost productivity of 0.62 (95% CI: 0.18, 1.05), 0.96 (95% CI: 0.64, 1.29), and 0.64 (95% CI: 0.27, 1.01) hours lost due to absenteeism, presenteeism, and unpaid work, respectively. Likewise, one additional point in the MFIS index represented a cost of CAD$95 (95% CI: 61, 128).

Those patients who had a relapse within the past 3 months lost 39 (95% CI: −0.07, 78.74) more hours due to absenteeism, 17 (95% CI: −24.47, −9.26) less hours due to presenteeism and showed costs of CAD$2851 (95% CI: −701, 6402) higher. Comorbidities, on the other hand, were not significantly associated with work productivity loss hours, but those pwMS having over three comorbidities showed a cost of lost productivity CAD$176 (95% CI: −849, 1201) higher than those with no comorbidities. Similarly, use of DMTs and quality of life utility, after adjusting for other variables, was not found to have a significant association with productivity loss.

Finally, employment status was associated with absenteeism and presenteeism, but not with unpaid work. Participants with a full-time job lost 36 (95% CI: 18.82, 52.79) and 18 (95% CI: 8.37, 27.89) more hours due to absenteeism and presenteeism, respectively, relative to those that were self-employed. Similarly, full-time job holders showed a cost of lost productivity CAD$2190 (95% CI: 1333, 3048) higher than self-employed workers.

## Discussion

This study characterizes productivity loss in a Canadian sample of employed pwMS including paid work productivity loss attributable to absenteeism and presenteeism and unpaid work productivity loss, and conducts a comprehensive monetary valuation of lost time. Overall, among a total work productivity loss of 60 hours in a 3-month period, presenteeism accounted for most (38%), followed by absenteeism (32%) and unpaid work loss (30%), of total loss. Assuming an 8-hour workday, our findings translate to approximately 2.5 days lost in a month. PwMS in our cohort lost approximately 7% of work time due to absenteeism and 5% due to presenteeism. Finally, lost hours represented an average total monetary cost of CAD$2480 over 3 months per MS patient when incorporating wage multipliers accounting for frequency of working with a team, team size, and influence on team function; and CAD$1848 when only using wages.

Two prior non-Canadian studies have measured productivity time loss using the work productivity and activity impairment questionnaire (WPAI). In the US study by Glanz et al.^30^ and the Australian study by Chen et al.^10^ the authors found that approximately 3.6% and 3.4% of productivity time loss was due to absenteeism and 11.9% and 10.8% due to presenteeism, respectively. Discrepancies with our findings are most likely explained by differences in the instrument used and variations in study subjects. A previous study found that WPAI provided the highest estimate of presenteeism (14.2 hours per 2 weeks) among four different instruments; while the health and labor questionnaire, using a similar direct hour estimation method to VOLP, provided the lowest presenteeism estimate (1.6 hours per 2 weeks).^31^ In addition, while our cohort is relatively young and at a very early stage of disease progression, those of Glanz et al.^30^ and Chen et al.^10^ included older patients who were approximately 12 years postdiagnosis. There are no available comparisons for unpaid work productivity loss, which was not included by Chen et al.^10^ and only provided as a mean percent activity impairment by Glanz et al.^30^

As for monetary valuations of lost time, existing costs attributable to absenteeism and presenteeism vary greatly across regions and MS severity levels as shown in a past systematic review and meta-analysis.^7^ Overall, current estimates of the value of lost productivity face two crucial gaps. First, they failed to account for unpaid work productivity loss, which based on our results is not a negligible component of productivity time loss. Other study findings that MS is more prevalent among women combined with greater unpaid work productivity losses for females^11^ could further affect total productivity loss estimations. Second, existing research in MS assigns a monetary value to time loss using reported personal income, which severely underestimates productivity loss as shown by our wage multipliers. The difference between the two cost approaches as shown for this study’s cohort at an early stage of disease progression is approximately CAD$632 per patient in a 3-month period, or an annual mean cost of CAD$2528. This illustrates how underestimated the overall burden of MS is when not accounting comprehensively for productivity losses beyond those of the MS employee alone.

We also explored statistically significant associations between productivity loss and a group of sociodemographic, clinical, and work-related factors. Contrary to previous findings in Germany,^11^ we found no association between gender and productivity loss, although females showed higher losses in each component, on average. Interestingly, work habits were also found not to be significantly associated with productivity loss outcomes. It could be that pwMS self-select into jobs that match their disability level, hence not significantly affecting their paid work productivity. The use of DMTs was also not significant, which is probably a reflection that DMTs tend to be more often used in people with more disease activity. As for relapses, consistent with published research,^12^ we found costs and absenteeism hours to be higher for those participants who experienced at least one relapse within the last 3 months. However, an opposite effect was found on presenteeism. That those with relapses showed lower productivity losses while working is likely driven by the fact that participants exhibiting relapses in our cohort are also younger and with a shorter disease duration.

The severity of MS as measured using EDSS was found to be associated with presenteeism, and unpaid work productivity loss, but not absenteeism. Several publications have studied the effect of EDSS on employment status, but evidence on its relationship with specific productivity loss outcomes is limited.^20^ Given the overall low severity of our cohort, participants might not need to take additional days from work, only experiencing reduced productivity while working.

The one factor consistently associated with all productivity loss outcomes was fatigue which is highly prevalent among pwMS,^32^ and has been consistently observed to be strongly associated with both leaving employment and hours lost.^20^ Notably, we also found that associations of productivity loss with fatigue were greater for presenteeism and unpaid work, confirming previous findings in the United States^30^ that fatigue could have a greater impact on regular daily activities than on paid work.

There are several limitations of this study. First, our productivity loss estimations and associations with key factors were developed using participants exclusively from the CanProCo study, with overrepresentation of patients at an early stage of MS (and even those who are asymptomatic), resulting in a cohort with low disease severity. Additional validation in other healthcare settings is therefore warranted to ensure generalizability. It is important to note that, given the low severity observed in our cohort, productivity losses in the general MS population are likely higher than our conservative estimates. Second, since we only used cross-sectional information, we were not able to examine changes in clinical factors and productivity loss over time. It is expected that, as the MS progresses, participants reduce their routine hours, and/or change jobs, further underestimating productivity loss estimates. Third, productivity loss is sensitive to the instrument used.^31,33^ Most previous studies in MS used the WPAI, which provides a higher presenteeism estimate as mentioned above and makes a comparison of our results with prior studies difficult. Future research on a standardized instrument for productivity loss will be informative.

Future studies could also use a longitudinal design to explore patterns of employment and productivity changes and to identify differences across MS phenotypes and a wider range of severity levels. Likewise, extending the study beyond employed individuals, the focus of this paper, will allow for the incorporation of costs of early retirement, work disability, and unemployment due to MS.

Overall, this study shows the importance of a comprehensive measure of productivity loss in determining the societal economic impact of MS, and the need to account for additional losses surpassing the wage loss of the person with MS. Effective interventions including workplace accommodations, psychosocial and pharmacological treatments, aimed at addressing the factors found to be associated with productivity loss, could enhance patient-oriented care, and potentially reduce the economic burden of MS.

## Supplemental Material

sj-docx-1-msj-10.1177_13524585211069070 – Supplemental material for Productivity loss among people with early multiple sclerosis: A Canadian study Click here for additional data file. Supplemental material, sj-docx-1-msj-10.1177_13524585211069070 for Productivity loss among people with early multiple sclerosis: A Canadian study by Elisabet Rodriguez Llorian, Wei Zhang, Amir Khakban, Scott Patten, Anthony Traboulsee, Jiwon Oh, Shannon Kolind, Alexandre Prat, Roger Tam and Larry D Lynd in Multiple Sclerosis Journal
