# Supplementary material for: Productivity loss among people with early multiple sclerosis: A Canadian study
Source: Mult Scler. 2022 Feb 9;28(9):1414–23. doi: 10.1177/13524585211069070 (PMC9260491; doi:10.1177/13524585211069070)
Supplement: sj-docx-1-msj-10.1177_13524585211069070 – Supplemental material for Productivity loss among people with early multiple sclerosis: A Canadian study [file sj-docx-1-msj-10.1177_13524585211069070.docx]

**SUPPLEMENTARY MATERIAL**

**PRODUCTIVITY LOSS AMONG PEOPLE WITH EARLY MULTIPLE SCLEROSIS: A CANADIAN STUDY**

Elisabet Rodriguez Llorian; Wei Zhang; Amir Khakban; Scott Patten; Anthony Traboulsee; Jiwon Oh; Shannon Kolind; Alexandre Prat; Roger Tam; Larry D Lynd

**S1. Supplementary Materials and Methods.**

1. **CanProCo Study**

**Inclusion Criteria:** Inclusion criteria for the CanProCo study included being between 18 and 60 years of age, having a confirmed diagnosis of radiologically isolated syndrome (RIS) or relapsing remitting-type of MS (RRMS) within 10 years of disease onset, or primary progressive-MS (PPMS) within 15 years of disease onset, and able to walk at least 100 meters independently or using a walking aid (Expanded Disease Severity Scale (EDSS) under 6.5).

**Ethics approval:** All participating sites obtained local research ethics board approval prior to study initiation.

- Providence St. Joseph’s and St. Michael’s Hospital Healthcare Research Ethics Board, St. Michael’s Hospital (18-325)
- Conjoint Health Research Ethics Board, University of Calgary (18-1853)
- Health Research Ethics Board, University of Edmonton (Pro00086907)
- Clinical Research Ethics Board, University of British Columbia (H18-03047)
- Comité d’éthique de la recherche du Centre hospitalier de l’Université de Montréal (18-293)

**Informed Consent:** Participants must speak and understand English or French to a reasonable degree and be able to independently provide written informed consent to participate in the study. Study related activities are not performed before completion of the informed consent document. Each participating site and funding body has executed required contracts and agreements outlining data access, storage and transfer. All participants have provided consent to publish their de-identified and aggregated data.

**Availability of data and materials:** At completion of the CanProCo study all participating sites will have access to final datasets. After study completion, study data will be made available to qualified external investigators through a data access request and review process.

1. **Productivity Loss**

For absenteeism, participants are asked about the days they were absent from work in the past 3 months because of their health (either due to physical, mental or emotional symptoms). Responses were converted to hours using collected information on participants’ regular working schedules.

Presenteeism was obtained as the percentage of time lost due to health while working, multiplied by the number of days worked (not lost due to absenteeism) in a 3-month period. The percentage of time loss while working follows the formula (a-b)/a, where a = the time taken to complete work in the past 7 days, b =the time that would have taken to complete the same work had the individual experience no health problems.

Lastly, unpaid work time loss in a 3-month period was measured by the hours needed from paid or unpaid help to complete unpaid activities such as housework in the past 7 days multiplied by 13 weeks.

1. **Assigning Monetary Value to Productivity Loss**

The rationale behind considering wage multipliers to calculate the costs of lost productivity is that ill-health may not only affect the productivity of the sick person but also that of other co-workers, based on team dependency and associated factors. Consequently, multipliers relating wage to marginal productivity allowed for the output loss to exceed that of the employee alone.

In the analysis, the average multiplier and wage were first calculated among the sample of pwMS who were employed and then applied homogenously to each patient to calculate their costs. This allows for variations in costs to be attributable to total productivity loss (time) rather than wages or multipliers.

**S2. Correlation matrix between fatigue, depression and anxiety indicators**

|  | **Fatigue** | **Depression** | **Anxiety** |
| --- | --- | --- | --- |
| **Fatigue** | - | - | - |
| **Depression** | 0.74 | - | - |
| **Anxiety** | 0.54 | 0.67 |  |

**S3. Comparison amongst employed pwMS depending on whether they had at least one productivity loss component missing.**

|  |  | **No missing component**  **(N=392)** | **Some missing component**  **(N=120)** | **p-value^1^** |
| --- | --- | --- | --- | --- |
|  | **Variable** |  |  |  |
| **Socio-demographic** | **Sex**, % Female | 279 (71%) | 85 (71%) | 0.94 |
|  | **Age (years)**, Mean (SD) | 38.96 (9.35) | 38.03 (10.02) | 0.35 |
| **Clinical** | **Severity^1^** |  |  |  |
|  | No disability EDSS 0 | 103 (26%) | 25 (21%) | 0.23 |
|  | Mild disability EDSS 1-3.5 | 278 (71%) | 89 (75%) | - |
|  | Moderate Disability EDSS 4-6 | 10 (3%) | 5 (4%) | - |
|  | **Time since diagnosis (years)**, Mean (SD) | 3.49 (2.72) | 2.90 (2.68) | 0.04 |
|  | **MS Type**, % by Phenotype |  |  |  |
|  | RRMS | 326 (83%) | 100 (83%) | 0.97 |
|  | PPMS | 18 (5%) | 9 (8%) | 0.21 |
|  | RIS | 28 (7%) | 1 (1%) | 0.01 |
|  | CIS | 20 (5%) | 10 (8%) | Ref. |
|  | **Current DMT users**, % | 219 (56%) | 65 (54%) | 0.74 |
|  | **Comorbidities, %** |  |  |  |
|  | 0 | 148 (38%) | 43 (36%) | Ref. |
|  | 1 | 109 (28%) | 30 (25%) | 0.55 |
|  | 2 | 63 (16%) | 21 (18%) | 0.71 |
|  | +3 | 72 (18%) | 26 (22%) | 0.42 |
|  | **Fatigue**, Median (Max-Min)^2^ | 22 (0-81) | 31 (0-75) | <0.01 |
|  | **Depression,** Median (Max-Min)^3^ | 4 (0-26) | 6 (0-19) | <0.01 |
|  | **Anxiety,** Median (Max-Min)^4^ | 3 (0-21) | 5 (0-21) | <0.01 |
| **Quality of Life** | **EQ-5D utility score,** Mean (SD) | 8.69 (0.91) | 8.33 (1.20) | <0.01 |
| **Work-related characteristics** | **Work habits,** % |  |  |  |
|  | Usually sits | 209 (54%) | 53 (50%) | 0.46 |
|  | Stand/walk | 117 (30%) | 35 (33%) | 0.59 |
|  | Light/Heavy Lifting | 64 (16%) | 19 (17%) | Ref. |
|  | **Employment status,** % |  |  |  |
|  | Full-time | 303 (77%) | 63 (53%) | <0.01 |
|  | Part-time | 49 (13%) | 35 (29%) | <0.01 |
|  | Self-employed | 40 (10%) | 22 (18%) | Ref. |

^1^ Differences between cohorts were tested using a t-test for continuous variables described using a mean and standard deviation, the Wilcoxon-Mann-Whitney test for variables described using a median, and a chi-square test for categorical variables.

Abbreviations: Expanded Disability Disease Scale (EDSS); Relapsing-remitting MS (RRMS); Primary-progressive MS (PPMS); Radiologically isolated syndrome (RIS); Clinical isolated syndrome (CIS)

**S4. Productivity Loss Components by Selected Variables, Hours**

| **Panel A. Productivity Loss Components by Disability Severity Level** | |
| --- | --- |
|  | \|  \| Total \| P \| A \| U \| \| --- \| --- \| --- \| --- \| --- \| \| Total \| 60 (107) \| 23 (52) \| 19 (52) \| 18 (62) \| \| EDSS=0 (Ref) \| 34 (65) \| 10 (25) \| 17 (46) \| 7 (30) \| \| EDSS>0 \| 69 (117) \| 27 (58) \| 20 (55) \| 22 (69) \| \| p-value \| 0.04 \| <0.01 \| 0.66 \| 0.03 \| |
| **Panel B. Productivity Loss Component by MS Phenotype** | |
|  | \|  \| Total \| P \| A \| U \| \| --- \| --- \| --- \| --- \| --- \| \| Total \| 60 (107) \| 23 (52) \| 19 (52) \| 18 (62) \| \| CIS (Ref.) \| 52 (66) \| 31 (46) \| 12 (29) \| 10 (25) \| \| RIS \| 23 (69) \| 13 (46) \| 6 (11) \| 5 (17) \| \| p-value \| 0.06 \| 0.3 \| 0.17 \| 0.24 \| \| RRMS \| 61 (109) \| 21 (49) \| 21 (56) \| 19 (64) \| \| p-value \| 0.57 \| 0.26 \| 0.08 \| 0.67 \| \| PPMS \| 100  (131) \| 53 (90) \| 10 (22) \| 37 (92) \| \| p-value \| 0.1 \| 0.01 \| 0.44 \| 0.19 \| |
| **Panel C. Productivity Loss Component by Sex** | |
|  | \|  \| Total \| P \| A \| U \| \| --- \| --- \| --- \| --- \| --- \| \| Total \| 60 (107) \| 23 (52) \| 19 (52) \| 18 (62) \| \| Female \| 63 (108) \| 24 (52) \| 21 (58) \| 19 (62) \| \| Male (Ref.) \| 51 (102) \| 20 (51) \| 15 (33) \| 16 (60) \| \| p-value \| 0.29 \| 0.49 \| 0.33 \| 0.70 \| |
| Notes: All statistics are hours lost and are calculated for those 392 pwMS with non-missing values for all three productivity loss components. Presented statistics are mean values (standard deviation in parenthesis) for each factor (severity, phenotype and sex) by productivity loss component. p-values are based on a t-test and compare each productivity loss category (total, P, A, U) between severity, phenotype and gender groups (base category is the variable level labeled as “Ref.”).  Abbreviations: Expanded Disability Disease Scale (EDSS); Presenteeism (P); Absenteeism (A); Unpaid work (U); Relapsing-remitting MS (RRMS); Primary-progressive MS (PPMS); Radiologically isolated syndrome (RIS); Clinical isolated syndrome (CIS) | |
